# The concept of medial pivot design from primary to revision total knee arthroplasty: a technical note

**DOI:** 10.1007/s00402-025-05798-9

**Published:** 2025-03-20

**Authors:** Patrick Sadoghi

**Affiliations:** Department of Orthopaedics and Trauma, Medical University of Graz, Graz, Austria

**Keywords:** Medial pivot, Primary total knee arthroplasty, Revision total knee arthroplasty

The concept of medial pivot (MP) in total knee arthroplasty (TKA) was patented in the 1990s and is driven by anatomic, fluoroscopic, and clinical studies [1–3, 8–10]. Its rationale is based on the fact, that the natural knee is more stable on the medial than lateral side. In approximation, it is medial a ball-in-socket, allowing for range of motion in all three planes around a medial center (pivot). The lateral aspect of the knee translates in flexion and shows discreet lateral lift off, due to the laxity of the collateral ligaments in flexion [1–3, 8–10].

Despite this, the kinematics of conventional knee arthroplasty have historically been based on an even gap in flexion and extension in neutral coronal alignment [4]. Majority of used conventional implants are cruciate-sacrificing without a post-cam mechanism and therefore indications for primary systems range from end-stage osteoarthritis of at least one out of three compartments to cruciate deficient knees and revisions without significant bone loss. However, in necessity for anterior–posterior (AP) stability with lack of integrity of the cruciate ligaments, a medial pivot system would guarantee sufficient conformity to address these cases [1].

In case of medio-lateral instability with a firm endpoint, conventional semi-constrained knees provide additional stability via the central post. However, this alters the biomechanics of the knee to a centrally guided ball in socket with some translation medially and laterally, if the knee has been properly balanced [6]. Non-physiological roll-back occurs here as a consequence, which is the lack of physiological posterior translation of the femur with progressive flexion with respect to the lateral compartment [1–3, 8–10] and no concept for medial pivot kinematics in these cases exists yet.

In case of the necessity for conventional hinged knee systems after multiple revisions without any ligament stability or in case of very complex primary cases, the coupling mechanism is located at the region of the tibial spine, resulting in an extra anatomical axis, pathological motion patterns including unphysiological roll-back, and no lateral lift off in flexion [5, 7]. This non-physiological kinematic movement leads to additional stress shielding around bony anchorage of stems and cones or sleeves [5, 7].

The obvious question is if a medial pivot concept, more akin to the natural knee kinematics, can be applied for medio-lateral or three-dimensional instability as well. Theoretically, the logical next step would be a medial pivot system in semi-constrained or full-constrained (hinged) revision total knee arthroplasty as hereby illustrated:

Figures 1, 2 and 3 demonstrate the patented concept from frontal, medial, and lateral [11]. The tibial and femoral components are mechanically coupled (hinged, full constrained) for pivoting around a medial center, made of titanium-coated cobalt-chromium molybdenum alloy, or ceramic, dependent on finite element and wear analysis. The mechanical coupling is affected in the area of the medial articulation surfaces via a first coupling element consisting of a ball and socket and in the area of the lateral articulation surfaces via a second coupling element consisting of a sliding plate or ball bearing with a different sagittal dimension from extension to flexion in order to allow for lateral lift off (a = b + 3 mm). Figures 4 and 5 demonstrate the axial view of the tibial plateau and femoral component, with the ball and socket mechanical coupling medial and the sliding plate coupling lateral and Fig. 6a, b demonstrate the medial coupling mechanism from medial and the lateral coupling mechanism form lateral.

**Figs. 1–3:**
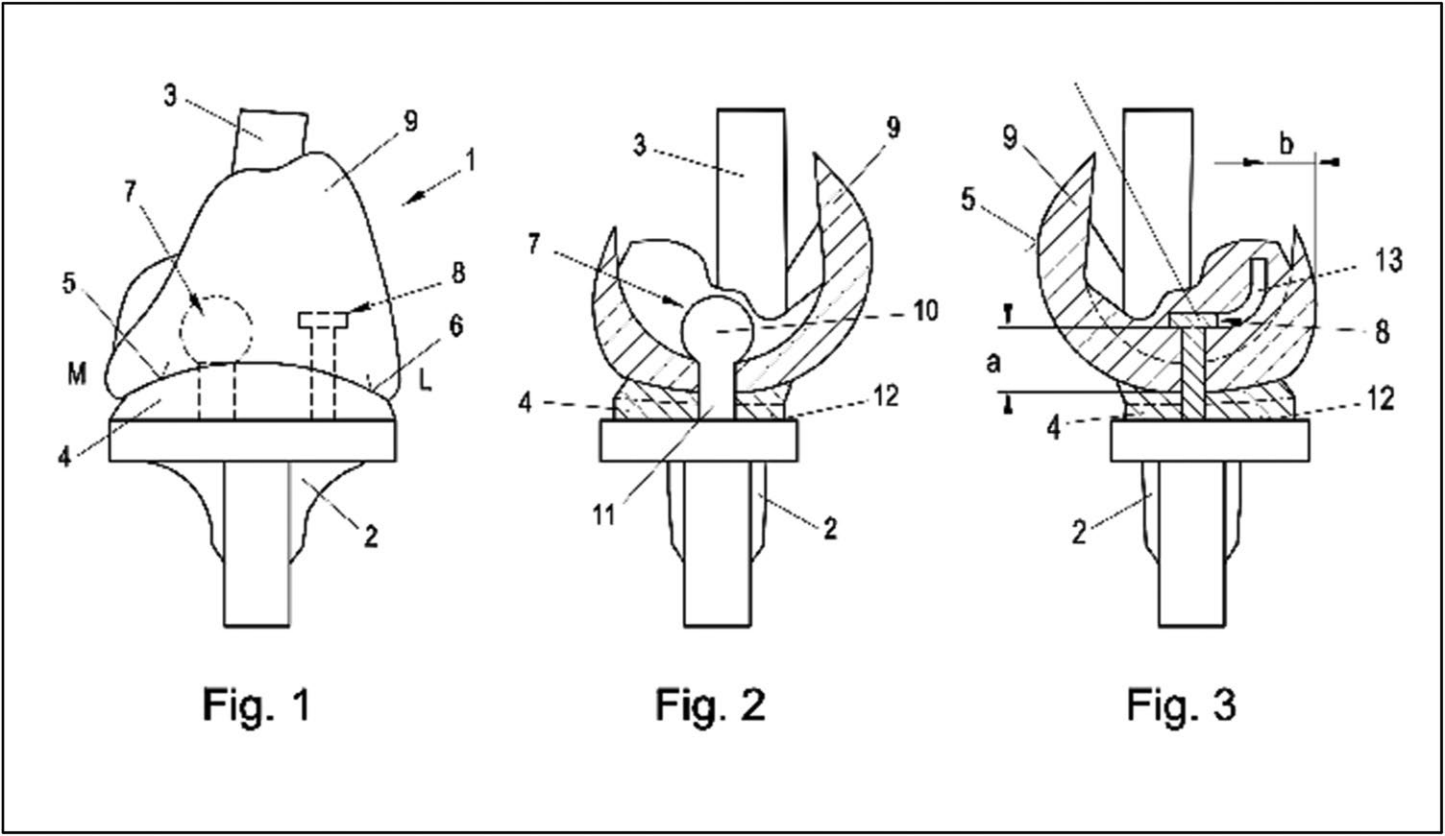
Illustration of the concept of a full constrained medial pivot revision total knee arthroplasty from frontal, medial, and lateral. The tibial and femoral components are mechanically coupled (hinged, full constrained) for pivoting around a medial center. 1: femoral component, 2: tibial plateau, 3: femoral stem, 4: inlay, 5: femoral component, 6: femoral component, 7: spherical gap for ball and socket joint, 8: sliding plate, 9: femoral component, M: medial, L: lateral, 10: ball on hinge, 11: coupling mechanism, 12: inlay, 13: flat gap for sliding plate, a = b + 3 mm (indicating lateral lift off in flexion)

**Figs. 4 and 5:**
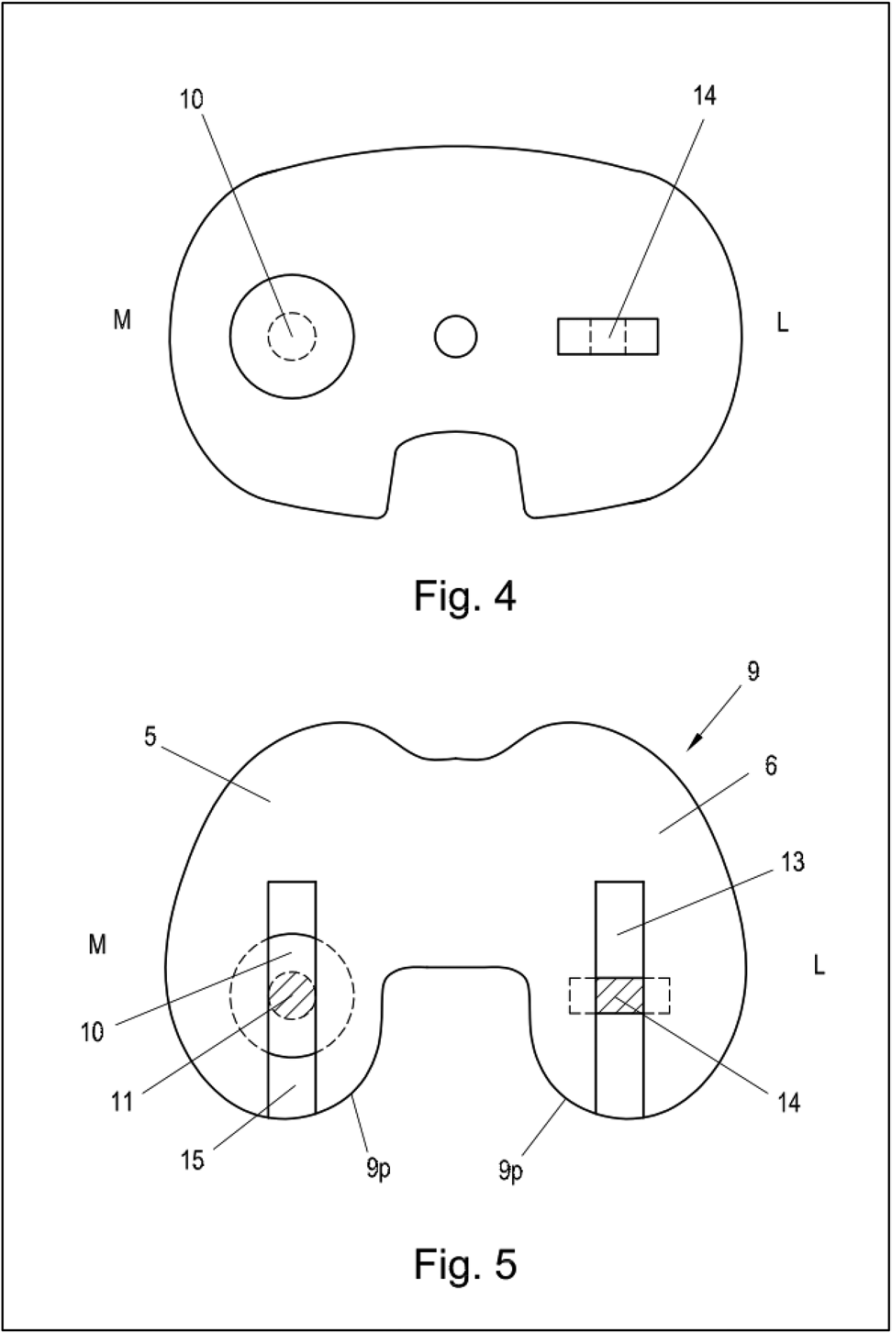
Illustration of the axial view of the tibial plateau and femoral component, with the ball and socket mechanical coupling medial and the sliding plate coupling lateral of the concept of a full constrained medial pivot revision total knee arthroplasty. M: medial, L: lateral, 10: stem and ball of the medial hinge, 14: sliding mechanism of the lateral coupling mechanism, 5, 6, 9, 9p (posterior): femoral component, 10, 11: spherical gap for ball and stem on femoral componenet, 13: canal for lateral coupling mechanism, 14: flat gap for sliding plate, 15: canal for medial coupling mechanism

**Fig. 6:**
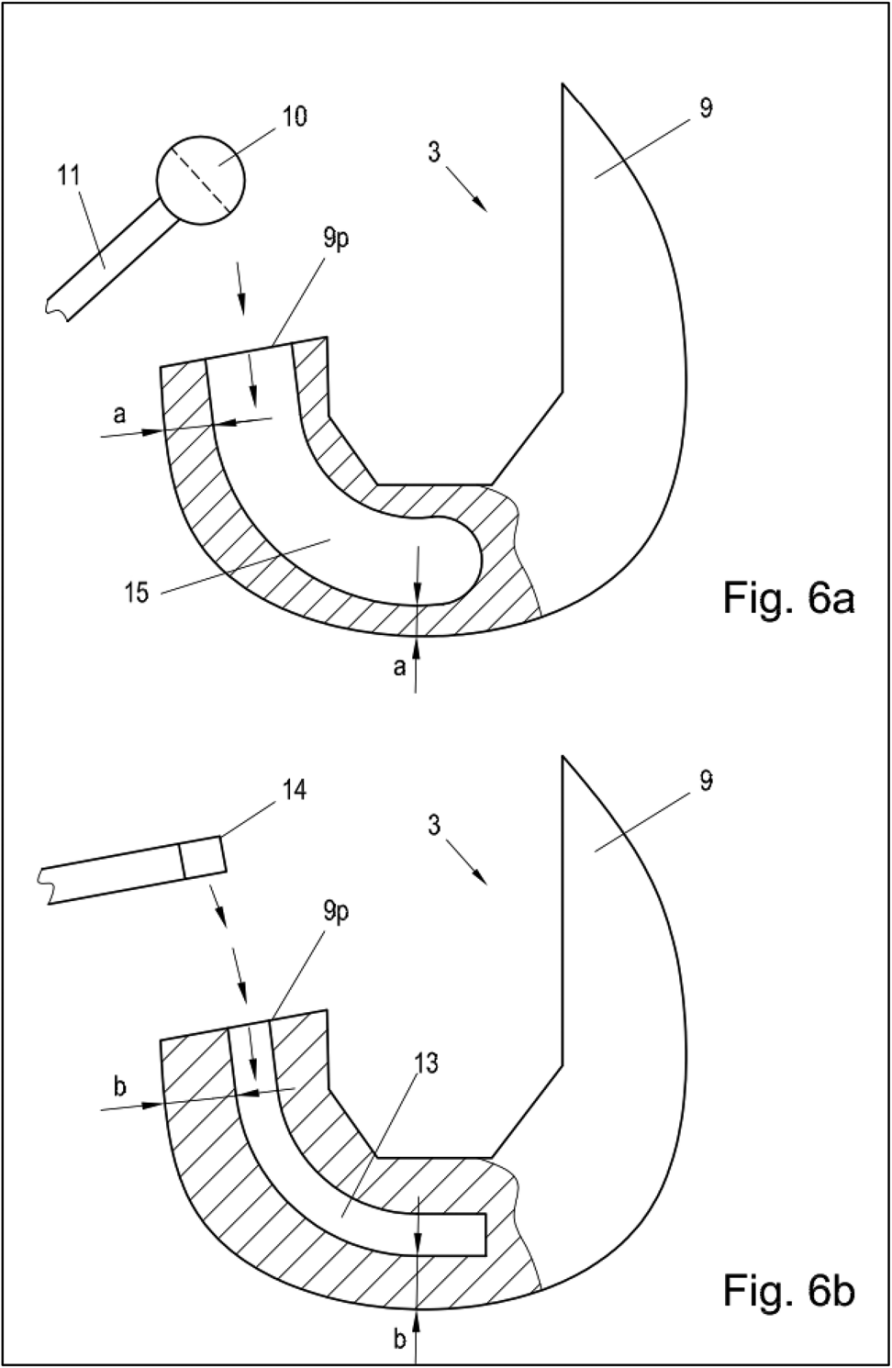
**a**, **b** Illustration of the medial coupling mechanism from medial and the lateral coupling mechanism form lateral of the concept of a full constrained medial pivot revision total knee arthroplasty. 3: femoral stem, 9: femoral component, 10: ball on medial hinge, 11: medial coupling mechanism, 13: flat gap for sliding plate, 14: sliding mechanism of the lateral coupling mechanism, 15: canal for medial coupling mechanism. a = b + 3 mm (indicating lateral lift off in flexion)

Potential limitations of the presented concept are difficulty to achieve adequate laxity in flexion. However, this was addressed by different diameters form extension to flexion of the lateral lateral articulation. In addition, implant failure due to the complex constrained mechanism including a medial and lateral aspect is more likely and decision on adequate material has yet to be found. Next, potential catching of the device has to be evaluated due to rotation and roll back of the lateral plateau.

With respect to translational research, there is a need for future in vitro trials, finite element analysis (FEA), and cadaveric studies to further elucidate this concept.

## Data Availability

No datasets were generated or analysed during the current study.

## Abbreviations

MP: Medial pivot
TKA: Total knee arthroplasty
AP: Anterior–posterior
FEA: Finite element analysis
